# Distally based anterolateral thigh flap: an underutilized option for peri-patellar wound coverage

**DOI:** 10.1007/s11751-018-0319-9

**Published:** 2018-10-01

**Authors:** Mikhail Bekarev, Abraham M. Goch, David S. Geller, Evan S. Garfein

**Affiliations:** Department of Orthopaedic Surgery, Montefiore Medical Center, 111 E 210th St, Bronx, NY 10467 USA

**Keywords:** Anterolateral thigh flap, Knee coverage, Peri-patellar wound, Knee defect

## Abstract

Wound coverage in the supra-patellar area presents a significant challenge for orthopaedic and reconstructive surgeons due to the need for preservation of knee joint function but the paucity of regional soft tissue flaps available. While many orthopaedic and reconstructive surgeons make use of the rotational gastrocnemius flap for coverage of peri-patellar defects, this flap has certain limitations. The goal of this study was to report a single-centre experience with the use of the distally based anterolateral thigh flap (ALT) and review the current literature on the use of the ALT for peri-patellar defects. In this report, both a single-centre experience using distally based anterolateral thigh (ALT) island flaps for supra-patellar wound coverage and the existing literature on this topic were reviewed. A systematic literature review was performed to assess the use of the ALT for peri-patellar wounds. Five patients with a mean age of 69 underwent a distally based ALT flap for coverage of peri-patellar defects. Four out of 5 flaps survived at the end of their respective follow-up. Based on this combined experience, the distally based reverse-flow anterolateral thigh island flap represents a useful but relatively underutilized option for appropriately selected supra-patellar wounds due to minimal donor site morbidity, multiple flap components, and predictable pedicle anatomy. The flap’s major weakness is its potentially unreliable venous drainage, requiring delay or secondary venous outflow anastomosis. Given the ALT flap’s favourable profile, the authors recommend consideration for its use when managing a peri-patellar coverage wound issue.

## Introduction

Peri-patellar wound coverage presents a significant challenge due to the paucity of regional soft tissue flaps and the need to preserve knee function. This is especially true for the superior half of the knee joint, where the gastrocnemius muscles do not typically extend. Whereas local tissue rearrangements and advancements have been reported to be effective for smaller defects, and free flaps have been used successfully to cover larger wounds, these methods have certain disadvantages for large wounds at or above the level at which the gastrocnemius reaches. The gastrocnemius flap has historically been the flap of choice for most orthopaedic and reconstructive surgeons for wounds at or below the level of the patella [1, 2]. The distally based, reverse-flow anterolateral thigh (ALT) island flap described in this report solves many of the problems left unaddressed by the gastrocnemius flap. It is an important treatment option that should be recognized by orthopaedic surgeons and included in the reconstructive surgeon’s armamentarium [3, 4].

## Materials and methods

An Institutional Review Board (IRB)-approved retrospective review of prospectively acquired data from authors’ database was performed. Inclusion criteria were reconstructive procedures of peri-patellar wounds performed between 2011 and 2015 with distally based ALT flaps. Any cases where other types of distally based flaps were used were excluded. Over the length of the study period, approximately 40 flap surgeries were performed for peri-patellar wounds. Five consecutive patients, 4 females and 1 male, were identified. Patient ages ranged from 50 to 85, with a mean age of 69. Distally based anterolateral lateral thigh island flaps were used for coverage of soft tissue defects created after wide excision of sarcoma arising in the peri-patellar or lower thigh region in 2 cases, or skin and soft tissue defects resulting from dehisced total knee arthroplasty wounds in 3 cases (Table 1). Intraoperatively, irrigation and debridement, and antibiotic spacer exchange were initially performed by an orthopaedic surgeon prior to flap dissection. Post-operatively, patients were maintained in either a knee immobilizer or a hinged knee brace and were mobilized non-weight-bearing for 2–6 weeks, depending on wound characteristics. Subsequently, patients were encouraged to begin progressive weight bearing as tolerated along with an instructed physical therapy regimen. Follow-up ranged from 1 to 36 months, with mean follow-up of 19 months.

**Table 1:** Patient demographics, flap parameters, and clinical and functional outcomes

Patient	Gender	Age	Diagnosis	Procedure	Flap dimensions	Wound closure	Rehabilitation protocol	Functional results	Complications	Follow-up period
1	Female	83	High-grade undifferentiated pleiomorphic sarcoma of right extremity status post-wide excision and reconstruction	Anterolateral musculofasciocutaenous flap	20 × 8 cm	Primary	NWB in Bledsoe brace × 6 weeks post-operatively, WBAT thereafter	Satisfactory. Right knee stiffness with 40 deg flexion, able to ambulate with a cane	Two non-healing wounds in incision area, subsequent thigh abscess at 2 months post-operatively, managed with IV abx and wound care, resolved	36 months
2	Male	85	Left knee osteoarthritis status post-left total knee replacement, complicated by infection	Anterolateral musculofasciocutaenous flap	19 × 10 cm	Wound vac, followed by STSG	NWB in Bledsoe brace × 6 weeks post-operatively, WBAT thereafter	Excellent. Mild left knee pain with 110 deg flexion, ambulates independently	Debridement and resection of patellar tendon at 2 weeks post-operatively, healed	11 months
3	Female	73	High-grade undifferentiated pleiomorphic sarcoma of left distal thigh, status post-wide excision with extensor mechanism reconstruction, complicated by infection and wound dehiscence	Anterolateral musculofasciocutaenous flap	17 × 10 cm	Primary	NWB in knee immobilizer × 2 weeks post-operatively, WBAT with no active knee flexion for 8 weeks, WBAT thereafter	Excellent. Left knee flexion 10–100 deg, able to ambulate with a cane	Partial flap congestion/necrosis at 1 week post-operatively, requiring debridement and STSG placement, healed	35 months
4	Female	56	Right knee osteoarthritis, status post-right total knee arthroplasty, complicated by infection and wound dehiscence	Anterolateral musculofasciocutaenous flap and medial gastrocnemius rotational flap	15 × 7 cm	STSG	NWB in knee immobilizer × 2 weeks post-operatively, WBAT thereafter	Excellent. Right knee flexion 0–100 deg, able to ambulate unassisted	Intraoperative venous congestion with delay of flap, successful coverage 10 days after. Partial flap necrosis post-procedure, requiring multiple debridements, healed	14 months
5	Female	50	Rheumatoid arthritis status post-right total knee arthroplasty, complicated by infection and wound dehiscence	Anterolateral musculofasciocutaenous flap	24 × 9 cm	STSG to flap inset area close to perforator	WBAT in knee immobilizer × 2 weeks post-operatively, WBAT thereafter	N/A	Persistent infection with subsequent right arterial occlusion distal to popliteal artery, treated with AKA 1 month post-index procedure	N/A

## Surgical technique

The site of the peri-patellar defect was measured. A skin paddle was designed over the anterolateral thigh in the standard position of an ALT free flap. The axis was drawn between the anterior superior iliac spine (ASIS) and lateral border of patella, which identifies the septum between the rectus femoris and vastus lateralis muscles, with the descending branch of the lateral femoral cutaneous artery (dLFCA) contained within. The proximal dLFCA and descending branch of the lateral femoral cutaneous vein (dLFCV) were identified, and proximal perforators into the skin paddle were noted. The proximal dLFCA and dLFCV were clamped, and perfusion to the skin paddle was assessed. At that point, perfusion was assumed to be supplied via the distal perforator arising from the superolateral genicular artery and forming a natural anastomosis with the distal lateral cutaneous femoral artery. If the skin paddle appeared congested or if arterial inflow was questionable, near-infrared laser angiography (SPY, Novadaq, Toronto, CA) was employed to assess the arterial inflow and venous outflow to the flap. If arterial inflow was suboptimal with the proximal pedicle clamped, the use of the distally based flap was aborted. If the venous outflow was suboptimal and the flap was congested, the flap was delayed for a period of 2–3 weeks to allow for intra-flap adjustment of venous drainage. A slip of vastus lateralis, tensor fascia lata, or both was harvested depending on need for soft tissue coverage. The distal perforator was then identified, and the flap was islanded by incising the skin and subcutaneous tissue just distal to this perforator. Once islanded, the entire flap was either tunnelled into the wound beneath subcutaneous tissue or inlaid into the wound by incising the intervening soft tissues. The donor site was closed primarily without tension when possible, or loosely approximated and overlaid with a split thickness skin graft (STSG) and negative pressure wound vacuum-assisted closure device where primary closure was deemed inappropriate.

## Results

Musculofasciocutaneous flaps were used in all cases, with flap sizes ranging from 15 × 7 cm to 24 × 9 cm. Each flap contained at least two perforators. Four out of 5 flaps survived to the end of their respective follow-up. The necrosed flap occurred in a patient with persistent peri-prosthetic infection and subsequent arterial occlusion distal to the popliteal artery, which required above the knee amputation of the ipsilateral extremity 1 month after the index procedure. One flap was found to exhibit intraoperative venous congestion and its harvest was delayed initially, with successful delayed coverage 1 week later. In one case, the medial head of the gastrocnemius muscle was used to cover a soft tissue defect not corrected by the anterolateral thigh flap alone. All flaps developed partial skin necrosis to varying degrees post-operatively, which was treated with wound care, negative pressure vacuum-assisted therapy, serial irrigation and debridement, and additional split thickness skin grafts as appropriate (Table 1). At the end of follow-up, 3 patients had minimal residual knee pain, functional knee joint flexion, and ambulated either unassisted or with a cane. One patient developed persistent knee stiffness, limited knee flexion to 40 degrees, and ambulated with a cane.

## Discussion

Peri-patellar and especially supra-patellar soft tissue defects represent a difficult reconstructive problem for orthopaedic and reconstructive surgeons due to paucity of soft tissue options in the region. Coverage of defects following oncological resection or salvage of infected knee prostheses requires healthy, well-vascularized soft tissue to maintain function and, ultimately, preserve the limb. In addition to restoring aesthetic appearance and skin integrity, orthopaedic and reconstructive surgeons must consider functional outcomes and the importance of restoration of the extensor mechanism of the knee joint. Local advancement and rotational flaps such as saphenous flaps or pedicled gastrocnemius flaps have been employed extensively for that role [5, 6]. However, drawbacks of these flaps include sensory deficits, possible need for STSG at the donor site, incomplete coverage for extensive tissue defects, decreased ipsilateral plantar flexion strength, and suboptimal overall functional outcomes [7–9]. These flaps are not well suited for subgroups of patients with loss of knee extensor mechanisms and need for tendon allograft. Alternatively, free flaps have been successfully used to address these shortcomings in coverage of complex peri-patellar defects; however, these flaps are associated with increased surgical time and require special facilities and microsurgical equipment [8]. The location of the recipient vessels represents an additional challenge for reconstructive surgeons intending to use a free flap for peri-patellar wound coverage.

First described by Song and Lou in 1984, the anterolateral thigh (ALT) flap quickly became a workhorse in myriad indications [10]. Available as free or pedicled flap, the ALT flap has been successfully used in the reconstruction of defects in various locations including lower/upper extremities, trunk, and head and neck [11, 12]. Advantages of the ALT flap include long pedicle, minimal donor site morbidity, its potential to be harvested using a 2-team approach, reliable flap anatomy, and multiple flap components (skin, fat, fascia, muscle, tendon, nerve), providing potential for tendon reconstruction and a neurovascular pedicle with sensate skin [13, 14].

The ALT is supplied by one to three perforators from the descending branch of the lateral circumflex femoral artery (LCFA) in more than 90% of cases. The descending branch was postulated to form a natural anastomosis with the lateral superior geniculate artery (LSGA) and/or profunda femoral artery [15]. Other anatomic variations might include a perforator arising from the deep femoral artery or a cutaneous perforator of the transverse branch of the LCFA. Shieh and Erba described at least four different types of perforator patterns arising from the main blood supply. Types I and III represent musculocutaneous and septocutaneous perforators, respectively, originating from the descending branch of the LCFA, whereas type II and IV perforators take route from the transverse branch of LCFA [16, 17]. The authors argued that type II and IV perforators preclude safe island flap harvest; however, subsequent reports have described successful island flaps using such perforators [18, 19]. Perforators differ in their course as well, and a hybrid musculoseptocutaneous perforator vessel has recently been reported as the most common vessel found radiographically, followed by strictly septocutaneous and musculocutaneous perforators [20].

The distally based anterolateral thigh island flap as first described by Wong was found to be a viable alternative to a free flap for local defect coverage [13]. The distal perforator was found to provide enough perfusion for the flap in most cases, with studies showing up to 80% of flow velocity of the original blood flow [15]. Other studies also suggested adequate peri- and post-operative blood flow by measuring velocity and oxygen perfusion of perforator flaps [21]. Recent cadaveric and imaging studies have established a relatively consistent distal vascular supply pattern for the distally based island ALT flap, with the proximal and distal perforators localized within 4–6 cm of the initial incision midpoint [18, 20, 22]. Others have suggested that anatomy is rather variable [23–26]. As such, clamping of the proximal vascular pedicle with either testing of skin bleeding or intraoperative laser angiography to ensure adequate perfusion from the distal perforator is always advised before committing to flap transfer; additionally, preoperative vascular mapping might be a useful adjunct to intraoperative imaging modalities [15, 20, 23, 24, 27, 28]. Near-infrared laser angiography using indocyanine green allows for assessment of perforator zones of perfusion prior to, and during flap harvest. This supplemental tool is an improvement over computed tomographic angiography (CTA) in that it provides dynamic information about perfusion without the radiation risk imposed by CTA. Additionally, it is superior to a unidirectional Doppler in that it can quantify relative tissue perfusion where the Doppler may locate small perforators that may be unable to support the flap [29].

Likewise, venous congestion is a problem characteristic of all reverse-flow flaps. This potential complication is described for the reverse-flow ALT and has been attributed to the resistance from venous valves seen with retrograde flow, as well as the possible tensioning and kinking of vessels that occurs during flap insetting. Acute venous congestion can be dealt with by flap delay, venous augmentation, vein grafting for bypass, or conversion to a free flap. Insidious development of venous congestion necessitates close monitoring with possible need for irrigation and debridement, negative pressure vacuum-assisted therapy, or venous supercharging [30]. This potential complication has also been reported to be successfully identified and prevented with preservation of a subcutaneous strip ranging from distal flap end to upper knee [18]. Alternatively, antegrade venous drainage through the microsurgical anastomosis with the proximal dLCFV was reported to effectively resolve venous congestion in at least one case [19]. Preventative venous supercharging during initial harvest represents an additional technique for minimizing venous congestion [31, 32].

The use of distally based anterolateral thigh flap represents an attractive option for reconstruction of peri-patellar soft tissue defects and has been reported in a few case reports and case series to date. Despite the potential functional and aesthetic benefits afforded by the use of this flap, it remains an underutilized option for wound coverage. To date, there exists a paucity of published literature describing the results of this flap for peri-patellar soft tissue defects. Pertinent results for each case series included are presented and discussed in brief (Table 2).

**Table 2:** Review of Current Literature

	Author	Cases	Skin closure	Flap parameters	Flap outcomes	Functional outcomes	Summary of authors’ recommendations
1.	Kimata et al. [3]	37	Primary 32/37 (86%) free flap 5/37 (14%)	14 × 15 cm to 21 × 35 cm	31/32 (97%) without any complication 1/32 (3%) with wound infection and marginal skin necrosis which resolved	Limitation to ROM 1/32 (3%) Muscle Weakness 10/32 (31%) Any sensory deficit 28/32 (87.5%)	Donor site morbidity is minimal and does not affect ADLs Morbidity correlates with damage to vastus lateralis and rectus femoris
2.	Kuo et al. [4]	38	All flaps < 8 cm in width closed primarily	10 × 4 cm to 26 × 12 cm	37/38 (97%) without any complication 1/38 (3%) severe infection with flap failure	Mild deficit in isokinetic concentric quad strength as compared to contralateral	Minimal donor site morbidity
3.	Gravvanis et al. [7]	1	1/1 closed primarily	Distally based ALT flap: 16 × 8 cm	1/1 flap survival, no complications	Excellent aesthetic and functional results at 3 months	More technically challenging, greater flexibility in size and shape than gastroc, better colour texture with less bulk, ease of re-elevation for repeat orthopaedic surgery, allows for early mobilization
4.	Akhtar et al. [8]	4	4/4 STSG	20 × 9 cm to 28 × 10 cm	1/4 (25%) distal marginal flap loss requiring I&D and grafting, 2/4 (50%) mild venous congestion which resolved within 3 days, 1/4 (25%) partial wound dehiscence with healing by secondary intention	No appreciable morbidity	Thin tissue, adequate length of vascular pedicle with flexible arc of rotation, early ambulation, cosmetically acceptable appearance and minimal donor site morbidity
5.	Song et al. [10]	1	1/1 closed primarily	Composite ALT 12 × 6 cm (with 14 × 10 cm fascia lata for patella tendon)	No complications	ROM 0-120 deg, normal strength at 30 months	Acceptable knee contour, one donor site for skin and extensor reconstruction, distally based ALT is difficult to dissect
6.	Gravvanis et al. [11]	2	2/2 closed primarily	Distally based island ALT flap 15 × 8 to 22 × 11 cm (1 case utilized a 3 × 18 cm fascia lata for patella tendon)	2/2 no complications	Excellent aesthetic and functional results at final follow-up including full knee ROM and no weakness	Minimal donor site morbidity, amplified blood perfusion, versatility, and large arc of rotation characterize ALT flap as an ideal pedicled flap
7.	Wong et al. [14]	8	8/8 closed primarily	ALT pedicled or free flap, sizes not included	1/8 (13%) distal flap necrosis requiring I&D and resuturing with healing, 2/8 (25%) with mild venous congestion which resolved within 1 week	ROM 0–90 (1/8) full ROM (7/8)	ALT flap offers versatility that is unparalleled by other options about the knee; however, its use requires flexibility regarding varying patterns of tissue transfer, skin paddle design and readiness to convert to free flap
8.	Pan et al. [15]	3	2/3 primarily 1/3 STSG	7 × 12, 7 × 16, 9 × 12 cm	No complications	Full ROM 1/3, satisfactory strength and ROM in 2/3	Advantages include: long pedicle, a sufficient amount of tissue, possible composite transfer with fascia lata for tendon reconstruction, and favourable donor site selection, without sacrifice of any major vessels or muscles
9.	Lu et al. [38]	518	Not provided	Pedicle length 12 cm, max dimensions 35 × 15 cm, chimeric ALT that can be thinned	Flap survival 496/518 (95.9%) Flap failure 22/518 (4.3%) Re-exploration for early signs of vascular compromise 72/518 (14%) Salvage 11/518 (2%), NPWT or local flap 3/518 (1%), amputation 8/518 (2%)	Not provided	ALT flap is authors’ preference in lower extremity reconstruction. In 5.5 per cent of planned anterolateral thigh reconstructions, perforators are unreliable, absent, or injured. The value of emergent backup plans may be underestimated
10.	Gao et al. [22]	15	12/15 primarily (80%) 3/15 with STSG (20%)	5/15 proximal tibia reconstruction, 10/15 middle tibia reconstruction, 15 × 5 cm to 30 × 12 cm free ALT using reverse descending LCFA as recipient artery for the contralateral ALT (mean flap 19.3 × 8.7 cm, mean pedicle 11.8 cm)	14/15 no complications (93%), 1/15 partial distal flap necrosis (7%) which healed by secondary intention	Not provided	Advantages of authors’ technique include a single-step procedure, availability of a large flap, a long vascular pedicle, large-calibre vessels, suitable flap thickness for coverage, and satisfactory flap appearance. Disadvantages include a complex surgical procedure, need for microsurgical technique, risk of anastomosis failure, possible anatomic variation, possible partial flap loss, and large scars on both thighs
11.	Heo et al. [23]	1	1/1 primarily	Distally based ALT 10 × 8 cm	1/1 no complications at 2 months	Functionally and cosmetically satisfactory	Free ALT flap has become the most ample and versatile flap for the reconstruction of skin and soft tissue defects because of its reliability, remarkably long pedicle, versatility, minor donor site morbidity and large potential size. Despite the anatomic variation of the perforators, the anatomy and dissection technique of the ALT flap have become well established
12.	Chen et al. [28]	1	1/1 primarily	18 × 10 cm	no complications	Ambulation without difficulty at 3 months	The ALT perforator flap based on the distal perforator can provide skin and soft tissue coverage around the knee with a satisfactory clinical outcome. The operative procedure is easy and reliable. This is an alternative option for soft tissue reconstruction around the knee
13.	Lin et al. [31]	18	13/18 primarily, 4/18 shoelace repair with progressive closure, 1/18 STSG	Distally based ALT with (*n* = 14) and without (*n* = 4) venous supercharging; Mean flap 21.4 × 8.8 cm (ranging from 12 × 6 cm to 27 × 12 cm). Pedicle 16.9 × 3.3 cm	Venous congestion in 4/4 (100%) of flaps without supercharging lasting 3-7 days, with partial flap loss in 2/4 (50%); Venous congestion secondary to anastomotic occlusion developed in 2/14 (14%) cases of the supercharged group. Early exploration with vein grafting resolved venous congestion in 1/14 (7%). Late exploration in the other resulted in total flap loss in 1/14 (7%)	Not included	Venous augmentation may improve the reliability of the distally based ALT flap. Preventive venous supercharge is suggested for the large, distally based ALT flap
14.	Yeh et al. [32]	4	4/4 primarily	Reverse-flow ALT without supercharging 12 × 6 cm to 20 × 10 cm, pedicles 8–16 cm	Venous congestion in 4/4 with resolution in 3–5 days in 2/4; 2/4 partial flap necrosis with I&D and STSG or local flap, all flaps eventually healed	Final ROM 0-120 at 8 years in 1 case, full ROM in another, no reported outcomes in 2/4	Reverse-flow ALT flap has versatile functions and limited donor site morbidity as seen in the conventional ALT flap. It is another option for soft tissue reconstruction around the knee and proximal lower leg; however, more reliable application of reverse-flow ALT must be based on smaller flap design or antegrade venous supercharge to reduce risk of venous congestion
15.	Nosrati et al. [34]	5	4/5 primarily, 1/5 STSG	121 cm^2; 2 pedicled and 3 free flaps	1/5 recipient site wound dehiscence, 1/5 donor site haematoma	4/5 return to preop functional status	A potential advantage of an ALT flap is the avoidance of utilizing a major knee flexor, such as gastrocnemius muscle. In addition, skin grafts are often necessary when the gastrocnemius muscle is used, whereas the ALT flap skin provides excellent resurfacing. The variety of ways in which ALT flap reconstructions can be performed suits the diverse tissue requirements of the entire lower extremity
16.	Demirseren et al. [35]	17	10/17 (59%) primarily, 7/17 (41%) STSG	Reverse-flow ALT perforator flap, largest flap 10 × 16 cm, largest pedicle 28 cm long	17/17 flap survival, 2/17 partial distal flap necrosis requiring I&D and local flap in one, STSG in another	Good aesthetic and functional results with full ROM in 17/17 by 3 months	Donor site defects < 10 cm can be closed primarily without complication. Although there are some critical points, such as planning of pivot point, inclusion of muscle cuff around the pedicle during dissection and prevention of the pedicle compression after the transfer, the reverse-flow ALT perforator flap is a good option, both aesthetically and functionally, for the reconstruction of soft tissue defects around the knee joint
17.	Liu et al. [33]	3	2/3 primarily (1/3 not mentioned)	Reverse ALT thigh island flap, 6 × 3 cm to 26 × 8 cm	1/3 distal and proximal medial marginal flap necrosis which healed secondarily by 1 month, 1/3 distal flap necrosis requiring I&D	No ROM restriction at 4 months, good functional recovery in 3/3	With a wide arc of rotation and sufficient skin paddle, the reverse anterolateral thigh island flap based on reverse flow is a good option for repairing skin defect around the knee; however, a staged or delayed operation might be considered in elevating a large flap
18.	Zheng et al. [36]	5	5/5 STSG	Chimeric ALT 22 × 12 cm to 6 × 4 cm	1/5 partial flap loss treated with I&D and STSG, 4/5 no complications	No patients experienced any difficulty in activities of daily living, and none of the patients suffered from knee extension lag. No patients experienced restrictions in climbing stairs	The chimeric ALT perforator flap, a novel variation of the standard ALT flap design, provides large tissue components that are versatile and valuable. This technique facilitates the harvest of various tissue components with maximal freedom, providing maximal flexibility to meet specific reconstructive requirements for large, complex, and irregular soft tissue defects in the extremities
19.	Erba et al. [18]	3	1/3 STSG	Distally based ALT 13 × 8 cm to 23 × 14 cm	1/3 marginal superficial necrosis at the distal edge, which healed by secondary intention, 1/3 need for explant and knee arthrodesis without graft complication 1/3 no complications	Full ROM and ability to ambulate	Distally based ALT flap is a safe and valuable alternative when approaching tissue reconstruction in the knee region. Identification of the perforator by Doppler analysis and routine preoperative angiography are highly recommended to identify perforators and their course up to the distal pivot point and to avoid critical Shieh Type II pedicled flaps. The preservation of a subcutaneous strip around the pedicle from the distal flap end to the upper knee further decreases risk of venous congestion

Chen et al. reported one case of a distally based ALT, 18 × 10 cm in size, for coverage of a fourth-degree burn in a distal thigh location, with the patient able to ambulate independently at 3-month follow-up [28]. The authors used a splint for 1 week post-operatively, with an early rehabilitation programme initiated promptly after. Similarly, Heo et al. published a case report of peri-patellar defect coverage with a distally based ALT. Preoperative computed tomographic angiography (CTA) was used to identify perforators, and an 8 cm × 10 cm flap was isolated with excellent functional outcome and no reported complications at 2-month follow-up [23]. Erba et al. described three cases with distally based ALT for defect closure after radical excision of malignant sarcoma of the lateral knee [18]. Their protocol included the initiation of physical therapy at 2 weeks post-operatively, with two patients ambulating by 2 weeks and achieving full range of motion at 2-month follow-up. All flaps survived and follow-up at 2 years was without complication. Additionally, Liu et al. reported on three cases of ALT island flap elevation to repair knee defects resulting from crush injuries [33]. These knee defects ranged from 3 × 6 cm to 26 × 8 cm. The authors described a standard ALT flap elevation technique, with the pedicle pivot point 6–7 cm above the knee joint. All three flaps survived; however, small areas of necrosis requiring debridement were noted at the margins in two out of three cases. Each series demonstrates minimal donor site morbidity with good functional outcomes.

Nosrati et al. reported on their experience with 5 cases of ALT flaps for coverage of knee defects as part of a larger series of 48 free or pedicled ALT flaps utilized for lower extremity defects [34]. Three free flaps and two pedicled flaps were used, with one of the pedicled flaps performed as a reverse-flow myocutaneous flap and the other as reversed vastus lateralis muscle-only flap covered with a split thickness skin graft. Two complications occurred, a recipient site wound dehiscence and a donor site haematoma, with all but one patient returning to preoperative functional status. Demirseren et al. presented their results on 17 reverse-flow distally based ALT flaps for reconstruction of peri-patellar defects and upper third of ipsilateral leg [35]. All flaps survived, and only two flaps developed partial necrosis at the distal ends, which improved after debridement. These slightly larger series demonstrate the flexibility that the ALT affords for reconstructive options.

Lastly, the chimeric ALT flap as described by Zheng et al. in their series provides for maximal versatility in reconstructing complex, irregular soft tissue defects. This flap is harvested using multiple tissue components such as muscle, fascia, and skin each supplied by a separate perforator off of the descending branch of the LCFA, providing for multiple spatially independent components to reconstruct composite defects [36]. The five lower leg defects repaired from their series of 22 patients ranged from 12 × 18 cm to 35 × 43 cm with complete flap survival in 4 and partial skin graft loss in one. At mean follow-up of 24 months, all wounds healed well with good contour in the reconstructed areas, no donor site complications, and no functional limitations.

## Limitations

While providing a significant soft tissue source for extensive coverage, ALT flap thickness can be a disadvantage, necessitating future debulking if the initial flap is thicker than the defect. Flap thickness has been extensively studied in Asian and Western populations, with greater thickness seen in the latter [20]. Although one-stage flap thinning techniques have been described, this carries additional risks of devitalizing the flap [37]. The subcutaneous fat thickness of the patients in this series was not such as to require thinning by either suction-assisted lipectomy or excisional techniques.

Additionally, the flap dissection can be laborious and complex with critical emphasis on the careful identification of a pivot point for the flap, as well as prevention of pedicle compression after transfer. The requirement for microsurgery is another potential limitation for this flap, as medical centres may lack the necessary equipment. Finally, the ALT has an uncommon but well-recognized possibility of anatomic variation that can provide for a difficult harvest in rare cases [22, 23, 35].

## Conclusions

The distally based reverse-flow anterolateral thigh island flap is a useful option for the coverage of peri-patellar wounds. It has the advantages of minimal donor site morbidity, reliable flap-harvesting approach, and multiple flap components. Despite these benefits, there are fewer than 100 cases reported worldwide to date, representing a significant underuse of this flap. In our opinion, the ALT flap represents an essential component of an orthopaedic and reconstructive surgeon’s armamentarium for addressing peri-patellar and especially supra-patellar wounds and should be included as a reconstructive ladder option when considering coverage options.
